# Prescribing Patterns of Evolocumab and Alirocumab in Patients With Familial Hypercholesterolemia: A Cross-Sectional Study Using the National Ambulatory Medical Care Survey, 2018–2019

**DOI:** 10.7759/cureus.93695

**Published:** 2025-10-02

**Authors:** Efeturi M Okorigba, Kehinde H Jinadu, Nneka Muoghalu, Emmanuel Adu, Ruthel S Rose, Akinyemi Akinwumiju

**Affiliations:** 1 Internal Medicine, West Virginia University, Morgantown, USA; 2 Internal Medicine, Spire Leeds Hospital, Leeds, GBR; 3 Public Health, University of Liverpool, School of Tropical Medicine, Liverpool, GBR; 4 Internal Medicine, University College Hospital, Ibadan, NGA; 5 Internal Medicine, St. John of God Hospital, Accra, GHA; 6 Internal Medicine, Kingston Public Hospital, Kingston, JAM; 7 Psychiatry and Behavioral Sciences, Progressive Psychiatric Services, Las Vegas, USA

**Keywords:** alirocumab, ambulatory care, evolocumab, familial hypercholesterolemia, pcsk9 inhibitors, prescribing trends

## Abstract

Background: Familial hypercholesterolemia (FH) is a hereditary lipid disorder that substantially elevates cardiovascular risk. Despite strong evidence and guideline recommendations, the real-world use of proprotein convertase subtilisin/kexin type 9 (PCSK9) inhibitors in FH remains uncertain.

Objective: This study aimed to evaluate national trends in PCSK9 inhibitor prescribing during ambulatory visits for adults with FH in the United States from 2018 to 2019.

Methods: We conducted a cross-sectional analysis of ambulatory care visits recorded in the 2018-2019 National Ambulatory Medical Care Survey (NAMCS), a nationally representative survey of office-based physicians in the United States. Eligible visits included adults (≥18 years) with a diagnosis of FH (International Classification of Diseases, 10^th^ Revision, Clinical Modification (ICD-10-CM) code E78.0). The primary outcome was prescription of a PCSK9 inhibitor (evolocumab or alirocumab), identified through medication codes. Patient demographics, insurance type, and physician specialty were examined. Survey weights were applied to generate national estimates, and logistic regression was used to explore the association between age and prescribing.

Results: Among the weighted total of 20,414,210 visits for adults with FH, only 69,672 visits (0.3%) included a prescription for a PCSK9 inhibitor. Prescribing was observed exclusively among female, White patients and occurred almost entirely in primary care settings. Patients receiving PCSK9 therapy were older than those not prescribed (mean 71.0 vs. 66.5 years, p=0.004). No prescribing was observed among males, racial/ethnic minority groups, or patients seen in surgical or medical specialties. In survey-weighted logistic regression, each additional year of age was associated with a 4% higher odds of PCSK9 prescribing (OR 1.04, 95% CI: 1.02-1.07, p=0.002).

Conclusion: PCSK9 inhibitor use in FH patients during U.S. ambulatory visits was rare, highlighting a persistent gap between evidence-based guidelines and clinical practice. Broader adoption strategies are needed to optimize lipid management and reduce cardiovascular risk in FH.

## Introduction

Familial hypercholesterolemia (FH) is a common, underdiagnosed, and untreated hereditary disease that is characterized by extremely high levels of low-density lipoprotein cholesterol (LDL-C) at birth, which result in accelerated atherosclerotic cardiovascular disease (ASCVD) and early coronary events [1]. It is estimated that around one in 250 people worldwide have heterozygous FH, and homozygous FH is still rarer, in about one in 300,000 to 1,000,000 individuals [2,3]. In the United States alone, this equates to over 1.3 million adults living with heterozygous FH, and only about 10% of them suffer a formal diagnosis [4]. FH is a significant public health problem despite some effective therapeutic strategies, due to underdiagnosis, less-than-optimal treatment, and a continued elevation of LDL-C in large numbers of patients [5].

In the past, the management of FH has been based on high-potency statins used alone or in combination with ezetimibe to lower LDL-C levels [6]. Although statins continue to be the foundation of lipid management, few FH patients attain recommended LDL-C targets with maximum tolerated doses [7]. Intolerance to statins, low effectiveness in high baseline LDL-C, or genetic resistance to standard therapy causes therapeutic gaps [8]. Consequently, there has been strong interest in novel pharmacologic approaches to reduce LDL-C and mitigate ASCVD risk in this high-risk population [9]. Evolocumab and alirocumab, which are proprotein convertase subtilisin/kexin type 9 (PCSK9) inhibitors, were among the most substantial innovations in lipid-lowering treatment over the last decade [10]. These sensitive human monoclonal antibodies prevent the degradation of LDL receptors mediated by PCSK9, and LDL-C clearance consequently increases drastically [11]. Multiple clinical trials supporting PCSK9 use have been established in patients with FH and include FOURIER (with evolocumab) and ODYSSEY OUTCOMES (with alirocumab): they have shown that PCSK9 inhibitors may decrease LDL-C (up to 60%) and major adverse cardiovascular event rates in high-risk patients considerably [12]. Accordingly, the American College of Cardiology (ACC) and the American Heart Association (AHA) have issued guideline recommendations stating that PCSK9 inhibitors should be used in adults with FH who fail to meet the LDL-C target on maximally tolerated high-intensity statin therapy combined with ezetimibe [13]. Further, previous studies have shown that despite its demonstrated efficacy, adoption of PCSK9 inhibitors in actual populations of FH has been slow and sparse [14]. Their high initial cost, extremely high levels of insurance authorization requirements, and a wide range of variability in prescribing practices to demographic and clinical subgroups are also considered as such [15]. The initial reviews showed that PCSK9 inhibitor prescription approval was as low as 20%-30%, most harmfully impacting minority and low-income patients [16, 17].

Such awareness of national trends in PCSK9 inhibitor prescription among FH patients is essential for enabling policy and addressing disparities, as well as for improving cardiovascular outcomes [18]. The National Ambulatory Medical Care Survey (NAMCS) provides a unique opportunity to study prescribing patterns on a national scale, as it represents actual clinical experiences in numerous specialties and demographic groups [19-22]. The main objective of the study is to estimate national trends in prescribing of PCSK9 inhibitors (evolocumab and alirocumab) during U.S. ambulatory care visits for adults with FH (International Classification of Diseases, 10^th^ Revision, Clinical Modification (ICD-10-CM) code E78.0 [23]) from 2018 to 2019, using NAMCS, and to describe differences by age, sex, race/ethnicity, payer, and visit specialty.

## Materials and methods

Study design and data source

This study employed a cross-sectional design using data from the 2018 and 2019 waves of the NAMCS [21]. NAMCS is conducted annually by the National Center for Health Statistics (NCHS) and uses a multistage probability sampling strategy to capture nationally representative information on visits to office-based physicians in the United States. The survey provides detailed data on patient demographics, clinical diagnoses, provider specialty, services delivered, and medications prescribed or supplied during the visit. Because prescribing of PCSK9 inhibitors occurs primarily in outpatient physician offices, NAMCS was considered the most appropriate dataset for this analysis.

Study population

The analytic cohort was restricted to visits made by adult patients aged 18 years and older. FH was identified using the ICD-10-CM code E78.0 [23], which is used within NAMCS to denote pure hypercholesterolemia and captures FH during this period. A visit was considered eligible if FH was listed in any of the three available diagnosis fields. Additionally, the primary outcome was whether a PCSK9 inhibitor was recorded during the encounter. PCSK9 prescribing was identified through the medication files when either evolocumab or alirocumab was coded using the Multum Lexicon drug identifiers (n16011 and n16020, respectively) [24]. Encounters without a diagnosis of FH or involving patients younger than 18 years were excluded.

Variables

The dependent variable was the prescription of a PCSK9 inhibitor. Independent variables included patient age in years, sex (male or female), and race and ethnicity (White, Black, Hispanic, and patients of other races/ethnicities). Insurance coverage was represented by the recoded PAYTYPER variable, which hierarchically classifies the primary expected source of payment into private insurance, Medicare, Medicaid/Children’s Health Insurance Program (CHIP), self-pay, or other categories. Provider specialty was captured using the NAMCS specialty classification, categorized into primary care, medical care, surgical specialties, and other. These covariates were selected based on prior evidence of demographic and system-level influences on cardiovascular medication use.

Statistical analysis

Survey weights, strata, and primary sampling units provided by NCHS were incorporated into all analyses to account for the complex sampling design and to generate nationally representative estimates of U.S. ambulatory visits. Descriptive statistics were first computed to characterize visits for patients with FH, stratified by whether or not a PCSK9 inhibitor was prescribed. Owing to the rarity of the outcomes of interest, with just one unweighted visit in which there was a prescription for a PCSK9 inhibitor, the analytical approach was essentially constrained. Further, the weighted proportions were compared across categories of age, sex, race/ethnicity, payment source, and physician specialty using survey-design-based F-tests for categorical variables and survey-weighted t-tests for continuous variables. Furthermore, given the small number of prescribing events, multivariable regression models with multiple predictors could not be reliably estimated. As an exploratory analysis, a survey-weighted logistic regression was fit with patient age (in years) as the sole independent variable to assess variation in prescribing patterns by age. Variance inflation factors (VIFs) for all covariates considered ranged from 1.02 to 1.45 (mean VIF = 1.15), indicating no multicollinearity concerns. After data preparation and restriction to adult visits with FH, all variables included in the analysis were complete, with no missing data. All analyses were performed using Stata version 18 (StataCorp, College Station, TX).

Ethical considerations

This study used publicly available, de-identified NAMCS data and therefore did not require institutional review board approval. NAMCS is conducted by NCHS under federal authority, and informed consent is obtained from participants at the time of data collection. Secondary analyses of this dataset are exempt from human subjects review under federal regulations.

## Results

Table 1 below summarizes the demographic and clinical characteristics of ambulatory visits for adults with FH in the 2018-2019 NAMCS, stratified by whether a PCSK9 inhibitor was prescribed. Weighted national estimates are presented, along with corresponding tests of association for each characteristic.

**Table 1:** Characteristics of ambulatory visits for adults with familial hypercholesterolemia (FH), stratified by PCSK9 inhibitor prescription—NAMCS 2018–2019 Data are presented as weighted frequencies (n) and percentages (%) unless otherwise specified. Age is expressed as mean ± standard deviation (SD). The overall sample included 20,334,537 visits without a proprotein convertase subtilisin/kexin type 9 (PCSK9) prescription and 69,672 visits with a PCSK9 prescription for patient age, sex, type of specialty, and race/ethnicity. For payment type, the analytic population was 19,613,503 visits (no PCSK9: 19,543,829; PCSK9: 69,672), as restriction to patients with FH led to the exclusion of categories associated with those without FH. Group comparisons were evaluated using survey-weighted t-tests for continuous variables and design-adjusted F-tests for categorical variables. Test statistics and corresponding p-values are reported in the table as F-test/t-test values. PCSK9 prescriptions include evolocumab and alirocumab. Statistical significance: ** p < 0.01 NAMCS: National Ambulatory Medical Care Survey

Characteristic	No PCSK9	PCSK9	F-test/T-test	p-value
Patient age(in years), (mean ± SD)	66.47± 11.10	71 ± 0	t= -3.11	0.004**
Sex (%)	-	-	F= 0.97	0.33
Male	10,615,454 (100%)	0	-	-
Female	9,729,082 (99.29 %)	69,672 (0.71%)	-	-
Payment type (%)	-	-	F= 0.01	0.98
Medicaid	549,141 (100%)	0	-	-
Private	405,398 (100%)	0	-	-
Missing/Unknown	18,589,289 (99.63%)	69,672 (0.37%)	-	-
Type of specialty (%)	-	-	F=0.08	0.88
Primary care specialty	13,731,394 (99.50%)	69,672 (0.50%)	-	-
Surgical care specialty	951,026 (0100%)	0	-	-
Medical care specialty	5,662,116(100%)	0		
Race/Ethnicity(%)	-	-	F= 0.02	0.93
White	15,667,665 (99.56%)	69,672 (0.44%)	-	-
Black	1,279,997 (100%)	0	-	-
Hispanic	2,887,632 (100%)	0	-	-
Others	509,242 (100%)	0	-	-

Among an estimated 20,414,210 ambulatory visits for adults with FH, only 69,672 (0.3%) included a prescription for a PCSK9 inhibitor. Patients prescribed PCSK9 inhibitors were older, with a mean age of 71.0 years compared to 66.5 ± 11.1 years among those not prescribed, a statistically significant difference (p=0.004).

By sex, PCSK9 prescribing occurred exclusively among female patients, with 69,672 (0.7%) of 9,798,754 (99.3%) female visits receiving a prescription. In contrast, none of the 10,615,454 (100%) male visits documented PCSK9 use. Consequently, no significant differences were observed by payment type (p=0.98). Visits with Medicaid coverage (549,141, 100%), private insurance (405,398, 100%), or unknown/missing payment information (18,658,961, 99.6%) did not include PCSK9 prescribing, except for a small fraction within the missing/unknown category (69,672, 0.4%).

Prescribing patterns were similarly limited across physician specialties (p=0.88). PCSK9 use occurred in 69,672 (0.5%) of 13,801,066 visits to primary care providers, with no prescriptions observed in surgical (951,026, 100%) or medical specialty visits (5,662,116, 100%). Moreover, race and ethnicity also showed no significant association with prescribing (p=0.93). PCSK9 use was confined to visits among White patients, representing 69,672 (0.4%) of 15,737,337 visits. No prescribing was observed among Black (n = 1,279,997, 100%), Hispanic (n = 2,887,632, 100%), or patients of other races/ethnicities (n = 509,242, 100%). In summary, these findings demonstrate that PCSK9 prescribing for FH in U.S. ambulatory care during 2018-2019 was exceedingly rare, occurring in less than 1% of visits, with utilization concentrated in older, female, White patients seen in primary care.

Table 2 below presents the results of a survey-weighted logistic regression analysis evaluating the association between patient age and the likelihood of PCSK9 inhibitor prescribing among visits for FH. Due to the extremely low frequency of prescribing events, age was the only predictor included in the model, consistent with the analytic limitations described in the Methods.

**Table 2:** Survey-weighted logistic regression of patient age and PCSK9 inhibitor prescribing among adults with familial hypercholesterolemia: NAMCS 2018–2019 Odds ratios (OR) and 95% confidence intervals (CI) are derived from a survey-weighted logistic regression model with patient age as the sole independent variable. Analyses account for National Ambulatory Medical Care Survey (NAMCS) sampling weights, strata, and primary sampling units. Statistical significance: ** p < 0.01.

Predictor	Adjusted Odds Ratio (OR)	95% CI	p-value
Patient age (in years)	1.04	1.02 – 1.07	0.002**

In exploratory regression analysis, patient age was significantly associated with PCSK9 inhibitor prescribing. Each additional year of age increased the odds of receiving a PCSK9 prescription by 4% (OR 1.04, 95% CI: 1.02-1.07, p=0.002). This finding suggests that, despite the overall rarity of PCSK9 use, older FH patients were more likely than younger patients to receive therapy. However, these results should be interpreted cautiously, as the analysis was constrained by the very small number of prescribing events, which limited adjustment for other demographic or clinical factors. Nevertheless, as a result of the smaller number of prescribing events, our findings should be interpreted with caution, given that the model might be overfit and unstable.

## Discussion

In this nationally representative analysis of ambulatory visits for adults with FH in the United States, the prescription of PCSK9 inhibitors was exceedingly uncommon. Across more than 20 million weighted visits during 2018-2019, only a small fraction documented the use of evolocumab or alirocumab. This limited uptake stands in sharp contrast to the robust efficacy demonstrated in clinical trials such as FOURIER and ODYSSEY OUTCOMES, which established PCSK9 inhibitors as powerful agents capable of lowering LDL-C by up to 60% and reducing the risk of major cardiovascular events in high-risk populations [12]. Moreover, the low frequency of prescribing observed in our study is consistent with earlier reports indicating slow adoption of PCSK9 inhibitors in clinical practice [14, 15]. Several barriers may help explain this finding, including the high cost of these medications, stringent insurance prior authorization requirements, and variability in clinician prescribing patterns [16, 17]. Our results further suggest that prescribing was concentrated in a narrow subset of patients, specifically, older, White women seen in primary care settings. This pattern aligns with previous concerns that sociodemographic disparities and system-level factors shape access to novel lipid-lowering therapies [14-17].

Importantly, although FH is both common and associated with markedly elevated ASCVD risk [1-5], many patients remain underdiagnosed and undertreated. The extremely low rate of PCSK9 utilization observed here highlights a missed opportunity to optimize therapy in a population for whom guideline-directed management includes PCSK9 inhibitors, even when LDL-C remains elevated despite statin and ezetimibe therapy [13]. These findings emphasize the continued need for policy interventions and clinical strategies to close treatment gaps and ensure equitable access to advanced lipid-lowering therapies for patients with FH.

Although the AHA/ACC strongly recommend PCSK9 inhibitors for high-risk FH patients who fail to achieve target LDL-C levels (<100) despite maximal statin therapy, adoption has been slow. PCSK9 inhibitors can significantly lower LDL-C and reduce cardiovascular risk [10], yet they remain underused. FH prevalence is estimated at one in 200 for heterozygous FH and up to one in 300,000 for homozygous FH. The condition responds poorly to conventional lipid-lowering therapy, often requiring a multifaceted approach [13]. The genetic variability of FH further complicates the definition of severe FH and the identification of high-risk individuals [13]. Evidence supports earlier initiation of PCSK9 inhibitors in severe FH, regardless of other lipid-lowering agents, and they are highly beneficial for high-risk individuals [12]. However, current AHA/ACC guidelines restrict their use to patients who have not achieved target goals. Despite these guidelines and supporting studies, this finding demonstrates a persistent gap between evidence-based recommendations and the limited clinical use of PCSK9 inhibitors in patients with FH.

In our findings, there was a lag in the utilization of the new PCSK-9 inhibitor recommendation due to, but not limited to, the following reasons: the cost-effectiveness of the medications is still being evaluated due to pricing concerns [7]. A knowledge gap amongst doctors who do not intermittently review the level of LDL-C because of overconfidence in the high-intensity statin prescription [7]. Also, it has been gathered that first-degree relatives of FH patients were not being investigated [6], which can generally lead to underdiagnosis of FH [6]. The invasive nature of the administration of PCSK-9 inhibitors also contributes to its limited widespread use [6]. Finally, the long-term safety of these compounds remains unclear, as data are still being evaluated from Phase 3 and 4 trials [6].

Several possible factors could explain the underutilization of PCSK9 inhibitors observed in our study. One key factor is the affordability of the drug. PCSK-9 inhibitors have been estimated to cost between US$12,000 and $14,000 annually as compared to “generic” statins, which cost less than US$100 annually [18]. PCSK9 inhibitors were more likely to be prescribed in patients with a high household income than those with low household incomes [14, 17]. Another barrier identified that limits the prescription of PCSK9 inhibitors is insurance. Several studies have revealed stringent prior authorization requirements, lack of insurance approval, and high out-of-pocket costs for co-pays as contributory factors to the low usage of the drug in the US population [15-17]. These medicines are not only costly but may also result in significant budget constraints for insurance payers. As a result, insurance providers may implement very inflexible policies and paperwork regarding coverage of these drugs, resulting in high rejection rates; it is reported that nearly two out of every three PCSK9 inhibitor prescriptions are rejected [15, 17]. In one study, several factors were identified as significantly determining the approval rates of these drugs by insurance providers. It was determined that higher approval rates were seen when the drugs were prescribed for older patients, prescribed by cardiologists, processed by specialty pharmacies, and prescribed to patients on government insurance plans [16]. Additionally, the long approval process time for these drugs was determined to negatively influence the availability of the drugs to patients. These factors highlight the interplay of various factors that determine how patients can get access to these drugs. However, even after drug approval, copayment remains a highly significant determinant of whether a patient would pick up the medication after drug approval [16]. Patient co-pay could range from $0 to $2822 per month supply [16]. In one study, it was determined that fewer than one in 10 patients with a $0 copay failed to fill their medication, whereas three out of four patients with a copay exceeding $375 did not pick up their prescription [16]. These continuous high out-of-pocket copay costs may deter patients from refilling their medications. Similar to our study, a previous study showed disparities related to sex and race among patients prescribed PCSK9 inhibitors, with racial minorities having a higher rejection rate of drug approval than whites [17]. The reason for this may be unknown and may warrant the need to explore further.

In both the 2016 International Atherosclerosis Society (IAS) consensus on severe FH and the AHA/ACC cholesterol guidelines, PCSK9 inhibitors are positioned as an important option for patients who remain far from LDL-C targets despite maximally tolerated statin and ezetimibe therapy. The IAS statement notes that in severe FH, characterized by markedly elevated untreated LDL-C or additional high-risk features, many patients do not reach safe cholesterol levels with standard drugs alone [13]. In such cases, PCSK9 inhibitors like evolocumab or alirocumab can reduce LDL-C by roughly 60%, with a substantial proportion achieving levels below 70 mg/dL. The AHA/ACC guidelines follow a stepwise approach, recommending high-intensity statin therapy first, then ezetimibe, and reserving PCSK9 inhibitors for those who remain above threshold values (generally >70 mg/dL in very high-risk patients). While both emphasize their efficacy, the U.S. guidance also takes into account cost considerations when deciding on long-term use.

Recommendations

Several studies have cited cost as a major deterrent to the use of PCSK9 inhibitors [15-17]. Every relevant stakeholder will need to be involved in the discussion, including clinicians, pharmaceutical companies, and the government, to formulate strategies to reduce costs. The government would have to work with pharmaceutical companies to explore ways to subsidize the medication. When compared with the cost of statins, the disparity is very wide [18]. The answer to reducing costs might lie in going generic. Furthermore, stringent prior authorization and a lack of insurance approval have been mentioned several times as barriers to use [15-18]. In some studies, nearly half of the patients prescribed PCSK9 inhibitors did not receive approval [16]. Advocacy to the insurance companies to see the need for the use of these medications. Lastly, some studies have suggested that the provision of samples to patients by clinicians can also help to expose them to PSCK9 inhibitors [16]. This may require more advocacy to encourage pharmaceutical companies to provide additional samples to clinicians, who are already very familiar with the use of statins [7]. Creating more visibility can help improve exposure to PCSK9 inhibitors.

Strengths and limitations

This study has several strengths. It uses data from the NAMCS, which provides nationally representative estimates of outpatient prescribing patterns across a wide range of physician specialties and patient demographics. By leveraging NAMCS, we were able to capture real-world clinical practice patterns, offering insights that complement trial-based efficacy data. Nevertheless, important limitations must be acknowledged. The most significant is the very low number of prescribing events, with only one unweighted PCSK9 prescription observed in the analytic sample. This limited the ability to evaluate associations beyond patient age and precluded robust multivariable adjustment for other demographic or clinical predictors. As a result, regression findings should be interpreted with caution. In addition, NAMCS data only included evolocumab and alirocumab during the 2018-2019 period, restricting our ability to assess longer-term prescribing trends or newer lipid-lowering agents. Therefore, future studies should build on these findings by using larger longitudinal datasets to track prescribing trends over an extended period and to incorporate newer lipid-lowering agents such as inclisiran. Research is also needed to better understand barriers to PCSK9 uptake at the patient, provider, and health system levels, including cost-related challenges and disparities in access across demographic groups. Such efforts could inform targeted interventions to improve guideline-concordant care and cardiovascular outcomes for individuals living with FH.

## Conclusions

In this nationally representative study of U.S. ambulatory visits, PCSK9 inhibitor prescribing for FH was exceedingly rare during 2018-2019. Utilization was observed only among a small subset of older, female, White patients in primary care. These findings highlight a substantial gap between guideline recommendations and real-world practice, underscoring the need for broader access and targeted strategies to optimize lipid management in FH. Thus, our findings, including the low utilization rate and the possible association with age, indicate a vital need for future, larger-scale studies designed with adequate power to definitively identify patient, provider, and system-level barriers to PCSK9 inhibitor prescribing in FH.
